# Editorial: Circulating biomarkers in prostate cancer

**DOI:** 10.3389/fonc.2024.1365353

**Published:** 2024-02-07

**Authors:** Masayoshi Nagata, Shigeo Horie, Yafeng Ma

**Affiliations:** ^1^ Department of Urology, Juntendo University Graduate School of Medicine, Tokyo, Japan; ^2^ Department of Advanced Informatics of Genetic Diseases, Digital Therapeutics, Juntendo University Graduate School of Medicine, Tokyo, Japan; ^3^ Ingham Institute for Applied Medical Research, School of Clinical Medicine, University of New South Wales, Liverpool, NSW, Australia

**Keywords:** prostate cancer, biomarkers, diagnosis, prognosis, liquid biopsy, circulating tumour cells

Prostate cancer is the fifth leading cause of cancer death worldwide and biomarkers with improved specificity and sensitivity are in urgent need for precise prognosis and diagnose. Circulating biomarkers, compared to biomarkers detected in solid tissues, consist of a diverse array of components found in the blood or urine, serving as diagnostic and prognostic indicators and aiding in the selection of effective drugs. These components include substances routinely measured in clinical peripheral blood analyses, such as blood cell constituents, electrolytes, and proteins for example albumin, alkaline phosphatase (ALP), and prostate cancer specific antigen (PSA). Although commonly utilized across various cancer types, these biomarkers are nonspecific and often serve solely as prognostic markers, lacking the clinical utilizations in personalized medicine. Recent advancements in liquid biopsy technology, allowing for the analysis of cancer-derived cells and molecules from peripheral blood, offer a less invasive and cost-effective alternative to traditional tissue biopsies. This Research Topic titled “*circulating biomarkers in prostate cancer*” comprises a collection of 2 systematic reviews, 3 (mini)reviews and 4 original articles, focusing on both conventional circulating biomarkers and biomarkers, defined by liquid biopsy, such as circulating tumour cells (CTC), cell free tumour DNA (ctDNA), extracellular vesicles(exosomes).

## Traditional circulating biomarkers

The geriatric nutritional risk index (GNRI), a conventional non-specific biomarker combining body weight and serum albumin levels, evaluates the nutritional status in cancer patients and proves valuable in predicting the prognosis in multiple cancer types (1, 2). Wu and Ye, through a meta-analysis, demonstrated the significance of pretreatment serum albumin as a circulating biomarker influencing the prognosis of urological cancer patients, and lower pretreatment GNRI predicts worse overall survival. Notably, PSA and its derivatives remain key traditional circulating biomarkers for early prostate cancer diagnosis, however PSA specificity is low. Ren et al. developed a predictive model for cancer positivity rates, incorporating various circulating biomarkers, including (f/T) PSA, blood inflammatory indicators hemoglobin to platelet ratio (HPR), neutrophils (NEUT), alongside clinical background (age) and multiparametric prostate MRI imaging PI-RADS (Prostate Imaging–Reporting and Data System) score. Augmenting image diagnosis with circulating biomarkers enhances diagnostic efficiency and accuracy.

## Biomarkers detected by liquid biopsy

**Figure 1:**
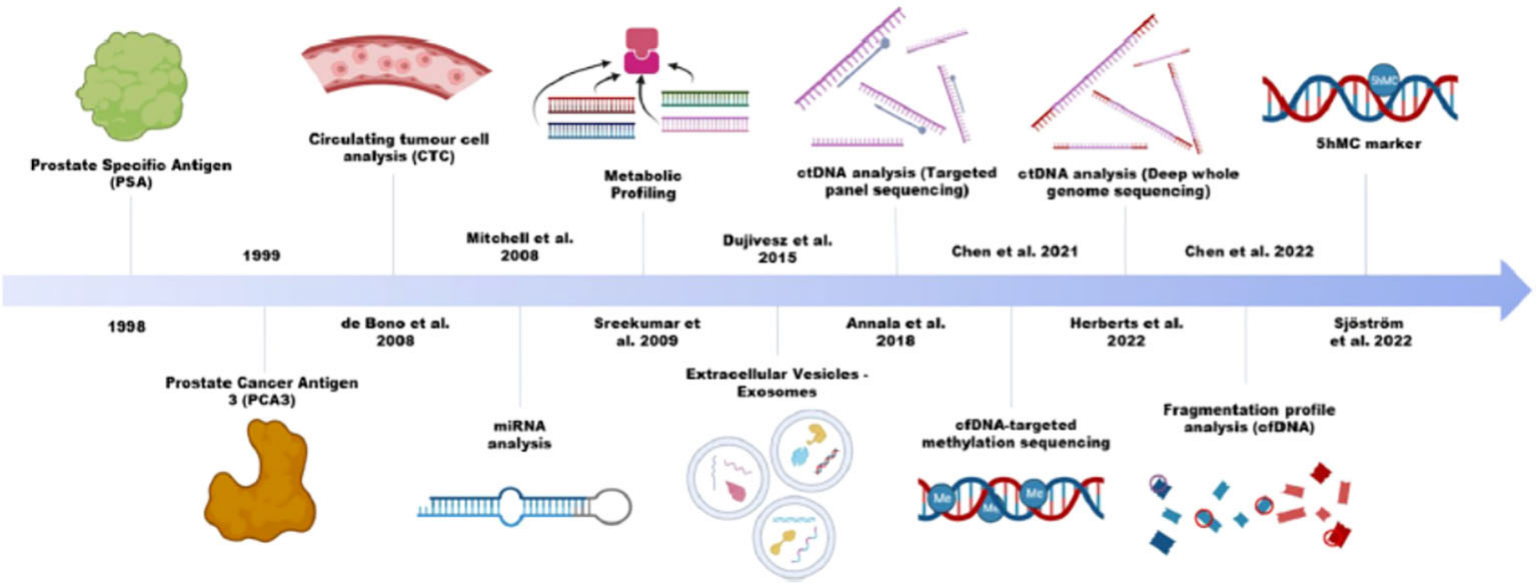
Application of various liquid biopsy technologies in prostate cancer.

Liquid biopsy demonstrates superiority in monitoring disease longitudinally and deciphering tumour evolutions. CTCs, ctDNA, exosomes (specially exosomal RNA), are commonly defined as liquid biopsy, offering insights into the genome and epigenome dynamics during prostate cancer progression. Lo et al. reviewed the genome-wide studies in prostate cancer focusing on cell free methylome (**Figure 1**) (3).

Molecular expression profiles and genetic abnormalities in CTCs, along with CTC count variations before and after treatment, provide powerful indicators of therapeutic efficacy (4). Furthermore, combining CTC measurements with clinical biomarkers, such as blood ALP levels and specific molecular expressions, enhances prognostic accuracy. In this Research Topic, Davies et al. captured pre-docetaxel treatment CTC from both metastatic castration resistant prostate cancer (mCRPC) and castration-sensitive PC (mHSPC), characterized CTC subtypes (epithelial, mesenchymal and EMTing) and gene expression analysis revealed the expression of docetaxel resistant gene ADAMTS1 and EMT transcription factors ZEB1 and SNAI1. Combination of total CTC number with PSA and ALP predict lack of partial response in mCRPC.

Homology recombination repair (HRR), the frequently altered DNA damage repair (DDR) mechanism in prostate cancer, involves genes like BRCA2. Poly ADP-ribose polymerase (PARP) inhibitors, such as olaparib and talazoparib, show antitumor activity in mCRPC with BRCA gene alterations (5, 6). Genomic testing for HRR gene mutations, especially using ctDNA, is crucial for guiding PARP inhibitor treatment. Liquid biopsy, particularly ctDNA testing, overcomes the limitations of tissue testing and allows longitudinal monitoring for emerging alterations and resistance mutations during disease progression. Catalano et al. reviewed current therapeutic indications in prostate cancer patients with DDR deficiency and provided the recommendations for germline and somatic -genomic testing in advanced PC and advantages of liquid biopsy in clinical utilities.

Dincman et al. emphasized the detection capabilities of ctDNA for abnormalities in various prostate cancer-related genes and highlighted the association of gene amplifications with disease features. Liquid biopsy, particularly plasma copy number analysis, aids in clinical decision-making for selecting appropriate therapeutic interventions during mCRPC treatment.

Long non-coding RNAs (lncRNAs), acting as regulatory transcripts of AR signaling and other important signaling pathways, play essential roles in prostate cancer development and treatment resistance. Urinary PCA3 (Prostate cancer antigen 3, a type of lncRNA) has been recognized as a biomarker for PC detection by U.S. Food and Drug Administration (FDA). In this Research Topic, Taheri et al. systematically reviewed the oncogenic roles of lncRNAs in prostate cancer pathogenesis and diagnosis and further emphasized the potentials and needs of early detection of prostate cancer with non-invasive methods, specially from blood.

The androgen receptor splice variant-7 (AR-V7) status, evaluated by both tissue and liquid biopsies, is highlighted as a potential biomarker for predicting drug resistance to enzalutamide and abiraterone, but not taxane chemotherapy in mCRPC (7–9). Zhao et al. performed a cumulative analysis on the relationship between AR-V7 status and the risk of CRPC, which only focused on the AR-V7 status in tissue biopsy.

Mo et al. explored Gene Expression Omnibus (GEO) database to discover potential prostate cancer associated biomarkers and identified 6 possible diagnostic markers, among which AOX1 could potential sever as a prognostic marker, further studies on its clinical value as a circulating biomarker are warranted.

## Summary

Ongoing research utilizing liquid biopsy for prostate cancer, characterized by minimal invasiveness and periodic analysis, is anticipated. Advancements in CTC and ctDNA detection technologies, coupled with whole genome sequencing, will likely continue, providing new insights into prostate cancer biology. These developments will contribute to the discovery of more effective treatments and targeted molecules for prostate cancer.

## Author contributions

MN: Writing – original draft, Writing – review & editing. SH: Writing – review & editing. YM: Writing – review & editing.
